# MXene-Enhanced Laser-Induced Graphene Flexible Sensor with Rapid Response for Monitoring Pilots’ Body Motion

**DOI:** 10.3390/mi16050513

**Published:** 2025-04-27

**Authors:** Xia Lei, Hongyun Fan, Yilin Zhao, Mian Zhong, Zhanghui Wu, Lin Li, Shouqing Li, Xiaoqing Xing, Jianhua Liu, Yibo Sun, Yong Jiang, Guogang Ren

**Affiliations:** 1 College of Aviation and Electronics and Electrical, Civil Aviation Flight University of China, Deyang 618307, China; leixia@cafuc.edu.cn (X.L.); 18731838513@163.com (H.F.); wu3732596zh@163.com (Z.W.); xingxiaoqing@cafuc.edu.cn (X.X.); ljh2583265@163.com (J.L.); 13190451939@163.com (Y.S.); 2 Faculty of Chemical Engineering, Kunming University of Science and Technology, Kunming 650500, China; zhaoyl@stu.kust.edu.cn; 3 School of Mathematics and Physics, Southwest University of Science and Technology, Mianyang 621010, China; 13158520181@163.com (L.L.); y_jiang@swust.edu.cn (Y.J.); 4 Civil Aviation Administration of China Academy, Civil Aviation Flight University of China, Deyang 618307, China; sqli@cafuc.edu.cn; 5 School of Physics, Engineering and Computer Science, University of Hertfordshire, Hatfield, Hertfordshire AL10 9AB, UK

**Keywords:** MXene/LIG, flexible wearable strain sensor, response, body motion

## Abstract

Flexible wearable strain sensors demonstrate promising application prospects in health monitoring, human-machine interaction, motion tracking, and the detection of human physiological signals. Although laser-induced graphene (LIG) materials have been extensively utilized in these scenarios, traditional types of LIG sensors are constrained by intrinsic limitations, including discontinuous conductive networks and electromechanical responsive hysteresis. These limitations hinder their applications in micro-strain detection scenarios. Consequently, enhancing the performance of LIG-based sensors has become a crucial priority. To address this challenge, we developed a novel MXene/LIG composite featuring optimized conductive networks and interfacial coupling effects through the systematic enhancement of LIG. The flexible strain sensor fabricated using this composite exhibits exceptional performance, including an ultra-low sheet resistance of 14.1 Ω, a high sensitivity of 20.7, a micro-strain detection limit of 0.05%, and a rapid response time of approximately 65 ms. These improvements significantly enhance electromechanical responsiveness and strain detection sensitivity. Furthermore, the sensor exhibits remarkable stability under varying tensile strains, particularly showing outstanding repeatability across 2500 cyclic tests. Notably, when applied to the pilot health monitoring scenarios, the MXene/LIG-based sensor demonstrates robust capability in detecting body movement signals such as micro-expressions and joint movements. This establishes a novel and highly effective technological solution for the real-time monitoring of pilots’ motion states during operational scenarios.

## 1. Introduction

Flexible wearable strain sensors have recently garnered considerable research attention for their applications in healthcare monitoring, motion tracking, and physiological signal detection [1,2]. Given that pilots, as a high-risk occupational group, play a critical role in aviation safety, the health conditions of pilots warrant close monitoring. These sensors demonstrate significant potential in capturing subtle physiological variations in real-time, thereby enabling effective monitoring of pilots’ physiological signals and health status and providing robust support for health management systems [3,4]. Nevertheless, several challenges remain in enhancing sensor performance, particularly regarding sensitivity improvement, response time optimization, and stability maintenance under operational conditions [5,6].

Among the diverse range of flexible sensing materials, laser-induced graphene (LIG) has emerged as a prominent candidate owing to its unique synthesis methodology and exceptional properties [7,8,9]. Through the process of laser irradiation of carbon precursors, such as polyimide (PI), LIG forms a three-dimensional porous graphene structure directly on the material’s surface [10,11]. This technique is characterized by its cost-effectiveness, single-step in situ fabrication, and exceptional environmental stability, which collectively enhance the material’s electrical conductivity, mechanical flexibility, and adaptability to various environments. Consequently, the LIG has become a critical approach for developing high-performance graphene-based sensors [12,13,14,15]. In comparison with traditional graphene synthesis approaches, the LIG technique demonstrates unparalleled simplicity and scalability, making it highly promising for applications in flexible wearable sensors [16,17].

Since Yakobson and colleagues pioneered the synthesis of three-dimensional porous graphene via CO_2_ laser direct ablation of PI in 2014 [18], significant advancements have been achieved in the preparation and application of LIG. Li et al. conducted a comprehensive review of the progress in LIG-based sensors for health monitoring, emphasizing various sensing mechanisms and applications [19]. For instance, Yang’s team developed a sweat sensor by integrating LIG with microfluidic technology to achieve high-sensitivity detection of uric acid and tyrosine in sweat. However, its one-minute response time limits the efficiency of dynamic signal detection. Additionally, the Torrente group fabricated a wireless mobile health device using an LIG-based electrochemical sensor capable of detecting cortisol in sweat within one minute [20]. Nevertheless, its prolonged signal processing time hinders real-time monitoring capabilities. Despite these achievements being remarkable in terms of sensitivity and multi-parameter detection, challenges remain, such as discontinuities in the conductive network and electromechanical response delays.

The performance of LIG is influenced by a variety of factors, such as the selection of carbon precursors, laser processing parameters, and dopant incorporation [21,22,23]. Among these factors, MXene materials have garnered significant attention due to their exceptional electrical conductivity, mechanical flexibility, and high specific surface area [24]. Although the intrinsic three-dimensional porous structure of LIG ensures superior conductivity, irreversible changes in interlayer contact resistance under cyclic strain often compromise the sensing stability. By incorporating MXene into carbon precursors for the fabrication of MXene/LIG composites, both enhanced conductivity and increased surface activity can be achieved [25]. Previous studies have demonstrated that adjusting the concentration of MXene significantly modulates key sensor performance metrics, including electrical conductivity, sensing sensitivity, and operational stability [26].

Against this backdrop, this study systematically investigates the synergistic mechanisms between MXene doping concentrations and LIG fabrication protocols for the development of a flexible sensor based on MXene/LIG composite architecture. Specifically, this study investigates the influence of variations in MXene concentration on the performance of flexible wearable strain sensors. Notably, this study features three key innovation points and contributions as follows:

1. Innovative modification of conventional LIG fabrication protocols enables the single-step fabrication of MXene-enhanced LIG. This advancement overcomes the inherent limitations of multi-step doping methodologies, offering a novel and efficient methodology for the production of high-performance flexible sensors.
2. Significant performance enhancement was achieved through MXene modification, characterized by a reduced sheet resistance (14.1 Ω), an expanded strain detection limit (0.05%), and a rapid response time (65 ms). These improvements substantiate the critical role of MXene in optimizing conductive networks and enhancing interfacial charge transfer dynamics.
3. Pioneering advancements have been made in the application of pilot health monitoring within the civil aviation sector. The MXene/LIG sensor exhibits remarkable capability in detecting subtle body movement signals. This innovation establishes an effective technological framework for real-time monitoring of pilots’ motion states during operational scenarios.

## 2. Materials and Methods

### 2.1. Preparation of LIG

**Figure 1:**
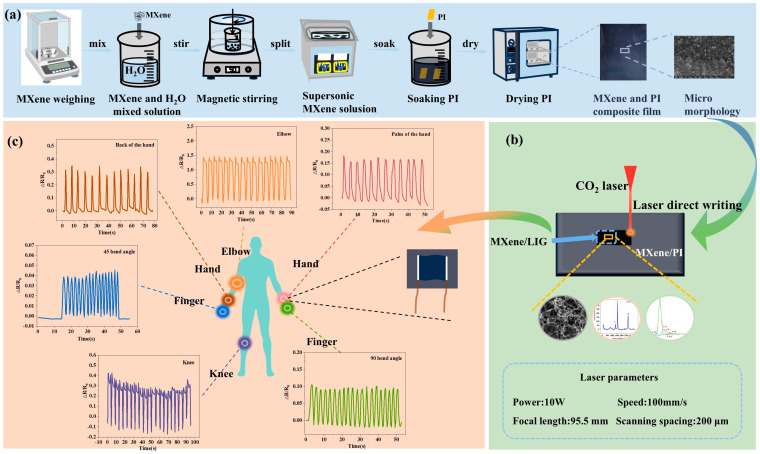
Fabrication process of the MXene/LIG-based flexible wearable sensor. (**a**) Treatment of PI with MXene solution; (**b**) laser direct writing; (**c**) assembly and application.

The experimental process for preparing the MXene/LIG composite is shown in Figure 1. Initially, 125 µm thick polyimide (PI) films (Shenzhen Jihongda Plastic Products Co., Ltd., Shenzhen, China) were immersed in MXene solutions of varying concentrations prior to undergoing the LIG process. The PI substrates were ultrasonically cleaned in deionized water for 10 min using an ultrasonic cleaner (Chun Rain Inc., Shenzhen, China), followed by drying at 50 °C for 30 min in a thermostatic drying oven (DHG-202, Shaoxing Subo Instrument Co., Ltd., Zhejiang, China). MXene solutions with concentrations of 5, 10, 15, 20, and 25 mg/mL were prepared by mixing predetermined masses of MXene powder (Sigma-Aldrich, St. Louis, MI, USA) with deionized water under magnetic stirring for 10 min. To ensure uniform dispersion, the solutions were further subjected to ultrasonication at 60 degrees Celsius for 20 min. The pretreated PI films were subsequently immersed in these MXene solutions for 30 min. Due to the hydrophilic nature of MXene and its abundant polar functional groups, stable adsorption layers formed on the PI surface via electrostatic interactions, van der Waals forces, and hydrogen bonding. After each immersion, the process was repeated three times to prepare partially black MXene-enhanced PI films, as shown on the right side of Figure 1. These samples were labeled as MXene_-x_/LIG, where x represents the concentration of the MXene solution (5–25 mg/mL). Subsequently, the MXene-enhanced PI films were secured on a three-axis motion stage and irradiated using a CO_2_ infrared laser system (Synrad P150, Novanta Corporation, Bedford, MA, USA) with fixed parameters: a laser focal length of 95.5 mm, wavelength of 10.6 μm, repetition rate of 20 kHz, pulse width of 100 μs, power of 10 W, scanning speed of 100 mm/s, and hatch spacing of 200 µm.

### 2.2. Material Characterization

In this study, the surface morphology of LIG was characterized using scanning electron microscopy (SEM; Thermo Scientiffc Helios 5 CX, Thermo Fisher Scientiffc Inc., Waltham, MA, USA). Energy-dispersive X-ray spectroscopy (EDS; Thermo Scientiffc Helios 5 CX, Thermo Fisher Scientiffc Inc., Waltham, MA, USA) was employed to analyze the elemental composition and spatial distribution in MXene/LIG composites. The positions and intensity ratios of the D-peak, G-peak, and 2D-peak in MXene/LIG were investigated using a Renishaw inVia confocal micro-Raman spectrometer (Raman; Renishaw Plc., Gloucestershire, UK). X-ray photoelectron spectroscopy (XPS; FEI ESCALAB Xi+, Thermo Fisher Scientiffc Inc., Waltham, MA, USA) was utilized to determine atomic species and their relative concentrations. Crystalline phase identification and peak intensity analysis were performed via X-ray diffraction (XRD; Rigaku Ultima IV, Rigaku Corporation, Akishima, Japan). Fourier-transform infrared spectroscopy (FTIR; Thermo field Nicolet iS5, Thermo Fisher Scientific Inc., Waltham, MA, USA) in the mid-infrared range was applied to detect functional groups within MXene/LIG. Real-time resistance measurements were conducted using a digital multimeter (RIGOL DM3058E, RIGOL Technologies Co., Ltd., Suzhou, China), while tensile strain testing was carried out with a universal testing machine (WNMC, Beijing, China).

## 3. Results and Discussion

### 3.1. Surface Morphology Analysis of MXene/LIG

**Figure 2:**
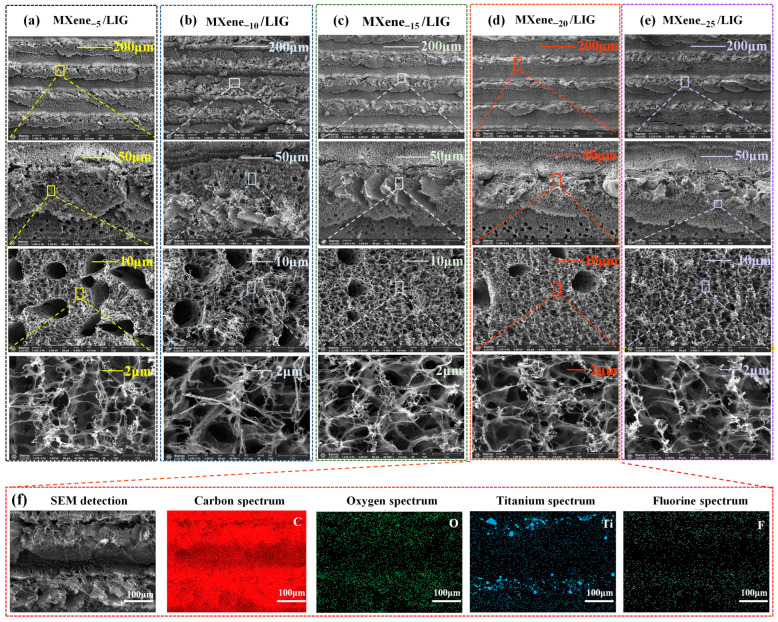
SEM characterization of MXene_−x_/LIG: (**a**) MXene_−5_/LIG; (**b**) MXene_−10_/LIG; (**c**) MXene_−15_/LIG; (**d**) MXene_−20_/LIG; (**e**) MXene_−25/_LIG; (**f**) EDS analysis of MXene_−20_/LIG.

In this section, optical spectroscopy characterization and analysis were performed on MXene_-x_/LIG composites synthesized via laser direct writing technology using PI films impregnated with MXene solutions of varying concentrations. Figure 2 presents SEM images of MXene_−x_/LIG for the evolution of surface microstructure. Figure 2a displays the SEM micrograph of MXene_−5_/LIG, revealing a relatively loose structure with comparatively larger pores (measured pore diameter range: 11.85–1.72 μm) and significant inter-pore voids, as quantified using Image View(V4.7.15144) software. Figure 2b illustrates MXene_−10_/LIG at higher magnification, revealing slightly reduced pore dimensions (8.25–1.5 μm) while maintaining structural dispersion. This phenomenon can be attributed to the onset of laser-induced thermal effects, which promote localized particle aggregation and moderate pore densification. Figure 2c demonstrates MXene_−15_/LIG, where further pore size reduction (7.19–1.38 μm) and enhanced particle aggregation are observed. The intensified thermal effects during laser irradiation likely enhance particle mobility, facilitating tighter packing. Figure 2d corresponds to MXene_−20_/LIG, exhibiting markedly denser structures with minimal pores (6.24–1.53 μm). At this concentration, the thermal effects induced by laser irradiation become more pronounced, with local heating promoting the flow of MXene particles, leading to their aggregation into larger agglomerates or massive structures and a further reduction in pore size. Figure 2e depicts MXene_−25_/LIG, characterized by nearly pore-free surfaces (3.7–0.95 μm), where laser energy induces extreme particle coalescence.

Statistical analysis of 30 randomly selected pores (excluding outliers) across Figure 2a–e yielded average pore diameters of 1.275 μm (standard deviation: 0.06457 μm), 1.521 μm (standard deviation: 0.07896 μm), 1.607 μm (standard deviation: 0.05954 μm), 1.84 μm (standard deviation: 0.0642 μm), and 1.412 μm (standard deviation: 0.05617 μm), respectively. MXene_−20_/LIG was identified as the optimal sensing material due to its homogeneous and densely packed architecture, which effectively balances enhanced electrical conductivity with controlled porosity. This structural configuration not only improves sensitivity, stability, and response speed but also circumvents the structural inhomogeneity observed at higher concentrations (e.g., 25 mg/mL). EDS analysis of MXene_−20_/LIG (Figure 2f) confirmed the presence of titanium, thereby verifying the successful integration of MXene into the LIG matrix.

### 3.2. Optical Spectrum Analysis of MXene/LIG

**Figure 3:**
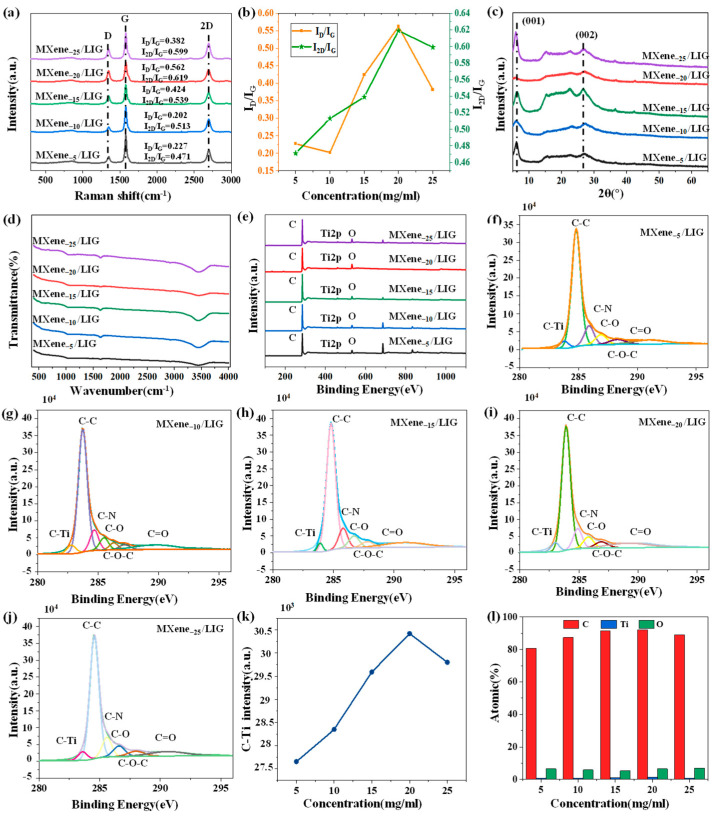
Spectral characterization of MXene_−x_/LIG: (**a**) Raman spectra; (**b**) calculation value of I_D_/I_G_ and I_2D_/I_G_; (**c**) XRD detection; (**d**) FTIR detection; (**e**) XPS detection; (**f**–**j**) MXene_−x_/LIG C1s subpeak and fitting; (**k**) MXene_−x_/LIG C-Ti peak intensity; (**l**) element content percentage.

Raman, XRD, FTIR, and XPS analyses were performed on the MXene_−5_/LIG, MXene_−10_/LIG, MXene_-15_/LIG, MXene_−20_/LIG, and MXene_−25_/LIG, with the results presented in Figure 3. Figure 3a displays the Raman spectra of MXene/LIG at varying concentrations, showing characteristic peaks: the D peak at 1344.86 cm^−1^, the G peak at 1578.21 cm^−1^, and the 2D peak at 2694.79 cm^−1^. Figure 3b illustrates the concentration-dependent trends of the intensity ratios I_D_/I_G_ and I_2D_/I_G_, which are associated with the graphene layer thickness, defect density, and structural quality. Lower MXene concentrations resulted in graphene with fewer defects and superior structural integrity, whereas higher concentrations led to an increase in I_D_/I_G_ due to MXene-enhanced defect generation. At a concentration of 20 mg/mL, graphene exhibited optimal structural stability, minimal defects, and a higher I_2D_/I_G_, indicating enhanced electrical conductivity and structural coherence.

Figure 3c illustrates the XRD patterns of LIG, which are utilized to analyze the crystal structure of LIG. Distinct (001) and (002) diffraction peaks are observed, indicating the presence of a well-defined crystal lattice. The variations in peak position and intensity reveal the influence of different MXene solution concentrations on the graphene crystal structure and interlayer spacing. Notably, the (002) peak is the most characteristic diffraction peak in graphene, directly reflecting the interlayer spacing. As the concentration of the MXene solution increases to 15 mg/mL, the intensity of the (002) peak gradually increases, indicating a reduction in the interlayer spacing and tighter stacking of graphene layers. This phenomenon may be attributed to the high MXene concentration, which promotes closer packing of the graphene layers. The (001) peak is typically associated with the fundamental arrangement of graphene’s crystal structure and its crystallinity. Its intensity and position can provide insights into the local orderliness and crystal structure of graphene. At a concentration of 25 mg/mL, the (001) peak exhibits the highest intensity, indicating superior crystal arrangement and enhanced crystallinity of the graphene material. Conversely, at a concentration of 20 mg/mL, the material may be in a transitional stage where the interlayer stacking and orderliness have not yet reached their optimal configuration, resulting in a lower intensity of the (001) peak.

Figure 3d demonstrates the FTIR spectrum, which elucidates the influence of varying MXene concentrations on the functional groups present on the graphene surface. Distinct absorption peaks at 1050 cm^−1^ (C–O), 1620 cm^−1^ (C=C), and 3450 cm^−1^ (–OH) confirm the hydrophilic characteristics of the material. Notably, at a MXene concentration of 20 mg/mL, the peak corresponding to hydroxyl groups becomes flattened, indicating a reduction in the density of hydroxyl functional groups on the graphene surface and an enhancement in hydrophobicity. This phenomenon suggests that, at this specific concentration, MXene and the PI film exhibit synergistic effects, resulting in a more complete LIG structure with minimized superfluous oxygen-containing hydrophilic functional groups. Conversely, when the MXene concentration is elevated to 25 mg/mL, the intensity of the hydroxyl peak increases once again. This may be attributed to structural heterogeneity induced by high-concentration MXene during the laser treatment process, leading to non-uniform distribution of functional groups.

Figure 3e illustrates the XPS test data, which primarily eliminates the variations in elemental composition on the graphene surface treated with varying concentrations of MXene solutions. Specifically, it focuses on the changes in binding energy and content of elements such as C, Ti, and O. The trend of elemental changes is displayed in a bar graph in Figure 3l. Figure 3f–j show the XPS peak splitting diagram of MXene_−x_/LIG C1s, which quantitatively analyzes the proportions of various chemical states, including C–C, C–O, and C–N bonds. These results not only reflect the oxidation of graphene-containing MXene but also confirm the presence of functional groups on its surface. Figure 3k further provides a comparative analysis of the C–Ti peak in MXene_−x_/LIG, revealing that the intensity of the C–Ti characteristic peak increases with the number of impregnation cycles. This trend confirms the formation of covalent bonding between MXene and LIG. This interaction facilitates the construction of a three-dimensional conductive network where MXene is embedded within the three-dimensional LIG framework.

### 3.3. Performance Evaluation of MXene/LIG-Based Sensor

**Figure 4:**
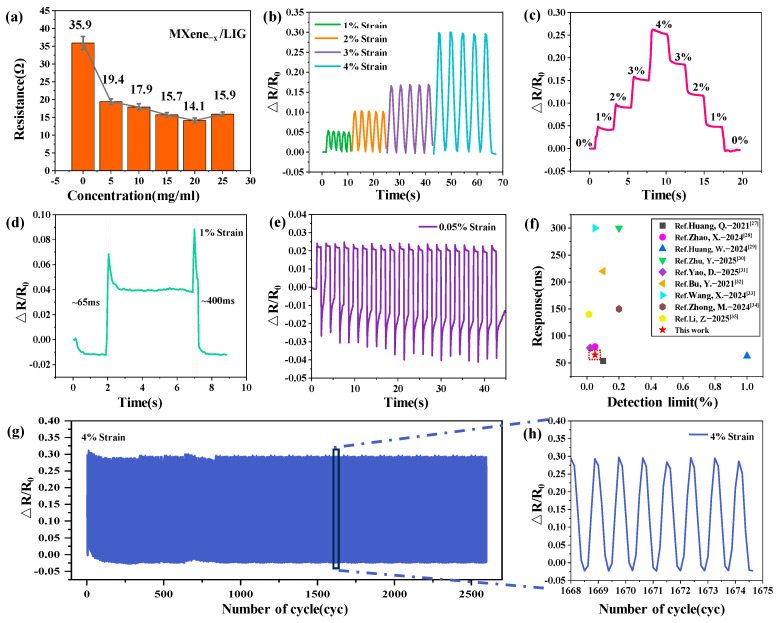
Performance valuations of the sensors: (**a**) Resistance characterization of the flexible wearable strain sensors based on MXene_−x_/LIG; (**b**) relative resistance change analysis during five cycles of stretching and releasing at strains of 1%, 2%, 3%, and 4%; (**c**) Continuous relative resistance change monitoring under sustained stretching and consecutive releasing at strains of 0%, 1%, 2%, 3%, and 4%; (**d**) time response assessment; (**e**) relative resistance change analysis for the minimum detection limit of MXene_−20_/LIG flexible wearable strain sensors; (**f**) comparative analysis of the minimum detection limit and time response between MXene_−20_/LIG flexible wearable strain sensors and other sensors; (**g**) Cycle repeatability evaluation of the flexible wearable strain sensors; (**h**) enlarged view of the cycle period from 1668 to 1675 in (**g**).

In the performance evaluation phase, electrical resistance measurements were initially conducted on MXene_−x_/LIG-based flexible wearable strain sensors fabricated with varying concentrations of MXene. As illustrated in Figure 4a, the sensor assembled with undoped LIG exhibited a baseline resistance of 35.9 Ω. Upon increasing the MXene concentration, the resistance of the MXene_−x_/LIG sensors demonstrated a decreasing trend, with recorded values of 19.4 Ω, 17.9 Ω, 15.7 Ω, and 14.1 Ω for concentrations of 5, 10, 15, and 20 mg/mL, respectively. However, at excessively high concentrations (25 mg/mL), an anomalous resistance increase to 15.9 Ω was observed, which may suggest potential structural saturation or agglomeration effects. Figure 4b presents the cyclic relative resistance changes (ΔR/R_0_) of the sensor under varying tensile strains (1%, 2%, 3%, and 4%) over time. The amplitude of resistance variation increases proportionally with increasing strain, reaching its maximum at 4% strain, where the ΔR/R_0_ response is most pronounced. This behavior suggests that progressive structural reconfiguration within the material under mechanical deformation—particularly microcrack propagation and conductive network disruption—leads to enhanced resistance modulation at elevated strain levels.

As demonstrated in Figure 4c, the variation in resistance exhibits a progressively increasing trend within the strain range of 0–4%. The magnitude of resistance change intensifies with increasing strain, indicating more pronounced conductivity modulation under higher mechanical deformation. This behavior validates the sensor’s superior sensitivity across extended strain ranges, which can be attributed to the strain-dependent structural reconfiguration of the conductive network. Figure 4d shows that upon applying a strain of 1%, the time response of the resistance change is rapid, particularly within the interval of approximately ±65 ms and ±400 ms. A swift time response indicates the sensor’s ability to promptly react during both the application and release of strain, offering an advantage for the detection of rapid movements. Figure 4e shows that at a strain of 0.05%, the variation in resistance is minimal. However, as the number of cycles increases, the change in resistance tends to stabilize. This indicates that under low-strain conditions, the sensor exhibits a weak response but maintains stable performance over prolonged usage. Figure 4f and Table 1 demonstrate that, compared to other sensors [27,28,29,30,31,32,33,34,35], the MXene/LIG-based flexible wearable strain sensor developed in this study exhibits superior performance in terms of the lowest detection limit and temporal response. The data points in the figure indicate that the sensor maintains excellent detection sensitivity even under small strains, with a rapid response time, outperforming other reference sensors. As shown in Figure 4g, with an increasing number of cycles, the resistance change remains relatively stable, particularly after approximately 1500 cycles, where the resistance fluctuation is minimal and consistent, indicating that the sensor possesses superior cyclic stability and is suitable for long-term applications. Figure 4h provides an enlarged view of the 1500–2500 cycle range from Figure 4g, showing periodic fluctuations in resistance at 4% strain, with the amplitude of resistance change remaining consistent. This confirms that the sensor can perform stable strain detection after multiple cycles of stretching and releasing, exhibiting outstanding repeatability and long-term stability.

### 3.4. Applications of MXene/LIG-Based Sensor

**Figure 5:**
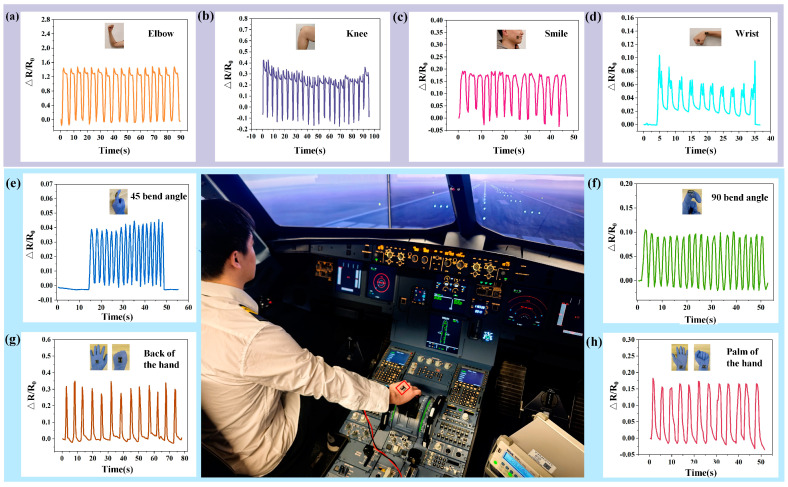
Relative resistance changes of the flexible strain sensor under various pilot-related motions. (**a**) Arm bending detection; (**b**) knee detection; (**c**) smile detection; (**d**) wrist bending detection; (**e**) Finger 45° bending detection; (**f**) Finger 90° bending detection; (**g**) back-of-hand stretch-clench detection; (**h**) palm stretch-clench detection.

To validate the practical applicability of the developed MXene/LIG-based flexible sensor, we innovatively applied it to pilot body movement monitoring. The developed flexible sensor was strategically attached to various body parts of pilots, including the wrist, fingers, arm, and knees, for comprehensive motion detection. As shown in Figure 5a, arm bending induced periodic resistance fluctuations with significant amplitude, revealing the impact of bending-induced surface deformation on the sensor’s performance. The bending motion subjected the sensor to tensile or compressive stresses, resulting in periodic variations in electrical resistance. Furthermore, the amplitude of resistance fluctuation varied correspondingly with variations in bending angle. Figure 5b demonstrates periodic resistance fluctuations during knee flexion-extension cycles, albeit with comparatively lower amplitude compared to arm movements, indicating distinct mechanical loading patterns. Figure 5c reveals subtle periodic resistance variations during facial expressions (smiling), which are associated with minute muscle contractions that induce micro-deformations. In Figure 5d, wrist articulation monitoring displays characteristic resistance waveforms generated by bidirectional motion-induced structural alterations in the sensor’s functional layer. Figure 5e–f reveal the resistance changes corresponding to finger bending at 45° and 90°, respectively. Compared to the 45° bending, the amplitude of resistance change is enhanced, reflecting the influence of the bending angle on the sensor’s surface morphology. Figure 5g–h show the resistance variations during transitions from hand back extension to a tight grip and from palm extension to a tight grip, both exhibiting pronounced periodicity. The resistance changes associated with the back-of-hand movements are attributed to increased muscle activity during a tight grip, while those related to palm movements involve significant contractions of the hand muscles, resulting in substantial fluctuations in resistance. These distinct responses highlight the sensor’s biomechanical relevance and electromechanical coupling efficiency.

## 4. Conclusions

In this study, we developed a flexible strain sensor based on MXene/LIG composite films, fabricated through solution immersion in conjunction with laser direct writing technology on PI substrates with precisely controlled MXene concentrations. The enhanced MXene/LIG sensor demonstrated exceptional performance characteristics, including ultralow resistance (14.1 Ω), an exceptionally low detection limit (0.05%), and rapid response time (~65 ms). Furthermore, it exhibited remarkable operational reliability during extensive cyclic testing while providing precise strain detection in practical scenarios, such as finger flexion and dorsal hand stretching. Systematic investigations revealed that both laser processing parameters and MXene concentration significantly influenced the pore architecture of LIG matrices, thereby establishing a direct correlation with the optimization of sensor performance. Notably, this study proposes a novel approach for enhancing MXene-based flexible sensors through synergistic material-process engineering, offering significant potential for application in aviation body movement monitoring systems, particularly in pilot health management.

## Figures and Tables

**Table 1:** Comparative analysis of MXene_−20_/LIG sensor relative to other sensing technologies.

Materials	Processing	Response Time (ms)	Detection Limit (%)	References
LIG	Laser direct writing	150	0.05	[22]
PDMS/PEI/CNT Sandpaper	Spray-coating	53.6	0.1	[27]
CNT/CB/TPU	Spraying carbon black	80	0.05	[28]
PAM/HA/MMT hydrogel	Dual physically cross-linked	62.5	1	[29]
PAAM/CS hydrogels	Surface coating	300	0.2	[30]
Hydrogel fiber	Introduction crack	78	0.02	[31]
MXene/paper	Dip coating	220	0.1	[32]
rGO/CNTs/TPU-TPU composite fibers	Dip coating	300	0.05	[33]
Nano-Silver-modified LIG	Laser direct writing	150	0.2	[34]
P-PDMS	Laser direct writing	140	0.0125	[35]
MXene/LIG	Laser direct writing	65	0.05	This work

## Data Availability

The original contributions presented in this study are included in the article. Further inquiries can be directed to the corresponding author(s).
